# Neuron and microglia/macrophage-derived FGF10 activate neuronal FGFR2/PI3K/Akt signaling and inhibit microglia/macrophages TLR4/NF-*κ*B-dependent neuroinflammation to improve functional recovery after spinal cord injury

**DOI:** 10.1038/cddis.2017.490

**Published:** 2017-10-05

**Authors:** Jian Chen, Zhouguang Wang, ZengMing Zheng, Yu Chen, Sinan Khor, KeSi Shi, ZiLi He, Qingqing Wang, Yingzheng Zhao, Hongyu Zhang, Xiaokun Li, Jiawei Li, Jiayu Yin, Xiangyang Wang, Jian Xiao

**Affiliations:** 1 Department of Orthopaedic Surgery, The Second Affiliated Hospital and Yuying Children's Hospital of Wenzhou Medical University, Wenzhou, China; 2 Molecular Pharmacology Research Center, School of Pharmaceutical Sciences, Wenzhou Medical University, Wenzhou, China; 3 Department of Molecular Pharmacology, Albert Einstein College of Medicine, Bronx, NY, USA

## Abstract

Therapeutics used to treat central nervous system (CNS) injury were designed to repair neurites and inhibit cell apoptosis. Previous studies have shown that neuron-derived FGF10 exerts potential neuroprotective effects after cerebral ischemia injury. However, little is known about the role of endogenous FGF10 in the recovery process after spinal cord injury (SCI). In this study, we found that FGF10 is mainly produced by neuron and microglia/macrophages, and its expression is increased after SCI. Exogenous treatment of FGF10 improved functional recovery after injury by reducing apoptosis, as well as repairing neurites via FGFR2/PI3K/Akt pathway. On another hand, inhibiting the PI3K/Akt pathway with LY294002 partially reversed the therapeutic effects of FGF10. In addition, small interfering RNA knockdown of FGFR2 suppressed PI3K/Akt pathway activation by FGF10 and abolished its anti-apoptotic and neurite repair effects *in vitro*. Furthermore, FGF10 treatment inhibited the activation and proliferation of microglia/macrophages through regulation of TLR4/NF-*κ*B pathway, and attenuated the release of pro-inflammatory cytokines after SCI. Thus, the increased expression of FGF10 after acute SCI is an endogenous self-protective response, suggesting that FGF10 could be a potential treatment for CNS injury.

Traumatic spinal cord injury (SCI) is a major cause of death and lifelong disability in the world, and over 250 000 people suffer SCI in the United States.^1^ The pathological process of SCI can be divided into two phases: (1) the primary injury characterized by direct local mechanical damage to the spinal cord at the time of injury, and (2) the secondary injury, which could possibly be counteracted using neuroprotective agents. Apart from local ischemia, other detrimental events such as local edema, focal hemorrhage, excitotoxicity and in particular, oxidative stress and post-ischemic neuroinflammation, contribute to prolonged secondary tissue injury after SCI.^2, 3^ These detrimental secondary events result in neuronal cell death or structural damage of surviving neurons, leading to physical and functional deficits.

Neurons are highly polarized cells that are composed of dendrites, which are tapered, shorter extensions to receive information and an axon, which is a thin, long hair-like extension to transmit information. Several studies have demonstrated the presence of neuronal death and axonal interruption around a primary lesion, which is the main obstacle preventing recovery from secondary damage.^4^ As a result of this, treatment of central nervous system (CNS) injury has revolved around repairing injured dendrites and axons and promoting their outgrowth. Cytoskeleton remodeling, such as microtubule assembly, occurs at the top of a growing neurite and is believed to be crucial for growth cone initiation and regrowth of injured neuritis.^5^ Microtubule dynamics give rise to a highly polarized morphology in neurons, as evidenced by a single axon and multiple dendrites.^6^ In recent years, it was reported that pharmacological treatment to stabilize microtubules promotes axon regeneration after SCI.^7^ In addition, it was reported that FGF13 acts as a microtubule-stabilizing protein to regulate neuronal migration and polarization in the cerebral cortex.^8^

Neurites of injured neurons in the adult CNS can seldom spontaneously regenerate in an inhibitory environment.^9^ Post-ischemic neuroinflammation is usually regarded as a deleterious factor to neurological function and leads to progressive deterioration of ventral horn motor neurons.^10^ The neuroinflammatory changes are attributed to microglia, the resident immunocyte in the CNS. Microglia are scavenger, which remove dead cells and are related to both elimination and maintenance of synapses for neural signal transduction.^11^ After injury, microglia are rapidly activated, undergoing morphological and molecular changes, which are related to neurotoxicity.^12^ As a result of the blood–brain barrier disruption,^13^ there are evidences that hematogenous macrophages contribute to secondary tissue damage in acute CNS injury.^14, 15^ However, the distinctions between microglia and macrophages in CNS have been elusive with the lack of discriminating marker.

When triggered by a stimulus such as direct mechanical trauma, activated microglia expresses high levels of Toll-like receptors (TLRs) in the CNS.^16^ Several studies have suggested that a wide array of TLRs, in particular TLR4, on microglia/macrophages can be further stimulated by secreted cytokines. This enhances a pro-inflammatory environment and exacerbates neuronal death and dysfunction.^17^ In a study performed using middle cerebral artery occlusion (MCAO) mice model, TLR4-deficient mice have less inflammatory response, contributing to minor infarct size after impact.^18^ These results link TLR4 signaling pathway and innate immunity with neuroinflammation triggered by ischemic injury.

Some endogenous factors released by the CNS that are induced by injury may also be beneficial recovery from the injury.^19^ However, the factors and associated mechanisms have not been fully investigated. The fibroblast growth factors (FGFs) are a family of cell signaling molecules released by various tissues that share a broad spectrum of biochemical and biological properties. FGF10 is a typical paracrine FGF and was originally cloned from rat embryos.^20^ Numerous articles have reported that innate FGF signaling promotes wound repair and tissue regeneration, regulates multiple organs development and maintains tissue homeostasis.^21, 22^ However, few articles have reported the role of FGF10 in CNS injury. A recent study reported for the first time that brain FGF10 is primarily produced from neurons and upregulated in a model for MCAO model.^23^ Exogenous FGF10 treatment ameliorated cerebral ischemic injury and reduced neuronal apoptosis as well.^23^ Nevertheless, whether there is a change in the expression of FGF10 and the signaling pathways activated by FGF10 after SCI have not been reported.

In this study, we aimed to explore the potential neuroprotective effects of FGF10 after acute SCI both *in vivo* and *in vitro*, as well as the mechanism by which it promotes neurite repair and prevents apoptosis. We further studied the mechanisms underlying the inflammatory response after SCI and the signaling pathways that mediate FGF10’s beneficial effects. Our results support that FGF10 may be a novel therapeutic intervention for SCI and pontentially could be useful for other traumatic CNS diseases.

## Results

### Neuron and microglia/macrophage-derived FGF10 increases activation of FGFR2/PI3K/Akt signaling after acute SCI

**Figure 1:**
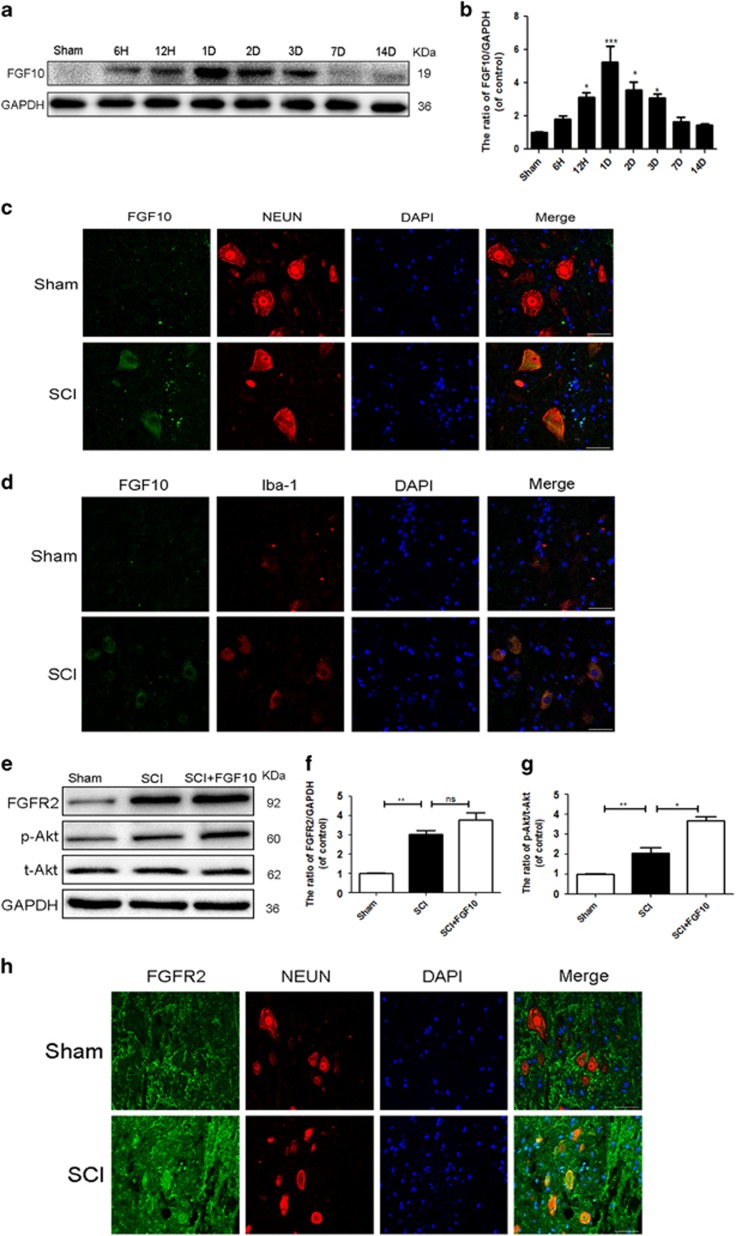
Neuron and microglia/macrophage-derived FGF10 increases with the activation of FGFR2/PI3K/Akt signaling after acute traumatic SCI. (**a** and **b**) Western blots and quantification data of FGF10 expression at several time points after acute SCI. (**c**-**e**) Double immunofluorescence of FGF10 and cellular markers for (**c**) neuron (NEUN) or (**d**) microglia/macrophages (Iba-1), in spinal cord tissue adjacent to lesion (scale bar: 50 *μ*m). (**e**-**g**) Western blots and quantification data of FGFR2, p-Akt and Akt in each group at 1 day after surgery. (**h**) Double immunofluorescence of FGFR2 and NeuN in sections from tissue at 1 day after SCI (scale bar: 50 *μ*m). Data represent the mean±S.D. Significant differences between the SCI and sham groups are indicated as *P<0.05, **P<0.01, ***P<0.001, *n*=5

In order to detect the expression of FGF10 after SCI, the T7–T10 level around the lesion epicenter of spinal cord tissue were excised. We assessed the influence of acute SCI on FGF10 protein expression at several time points from spinal cord tissue. Western blot analyses showed that FGF10 was significantly increased after acute SCI and peaked at 1-day post-operation (Figures 1a and b). Double immunofluorescence staining in spinal cord tissue adjacent to the lesion showed that FGF10 is mainly present in neurons and microglia/macrophages rather than astrocytes (Figures 1c and d, Supplementary Figure S1a). And double immunofluorescence for FGF10 and CD68, an anther marker of microglia/ macrophages, showed the same result (Supplementary Figure S1b). Thus, we hypothesized that FGF10 may have a role in neurons and microglia**/**macrophages. FGF10 mediates numerous biological responses by activating FGFR2/PI3K/Akt signaling in a paracrine manner. However, few articles have examined the expression of FGFR2 after acute SCI. Interestingly, we observed higher expression of FGFR2 and ratio of p-Akt/t-Akt after acute SCI by western blotting, and FGF10 treatment upregulated the expression of p-Akt, but slightly change the level of FGFR2 in SCI group (Figures 1e–g). Double immunofluorescence staining for FGFR2, as well as neurons (NEUN) showed that FGFR2 is significantly upregulated in neurons, similar to FGF10. The SCI group had increased FGFR2-positive puncta in neurons compared with sham group (Figure 1h). Furthermore, we found the increasing expression of other FGFs, such as FGF1, FGF2 and FGF7in SCI group, (Supplementary Figures S1c–g), which is consistent with other study for FGF1 and FGF2.^24^ However, our study first reported the increasing level of FGF7 after SCI, especially in acute stage, which may activate FGFR2 after SCI.

To determine whether FGF10 activated PI3K/Akt signaling is mediated by FGFR2, FGFR2 small interfering RNA (siRNA) was used to knockdown FGFR2 in PC12 cell before FGF10 treatment (Supplementary Figures S2a-c). FGFR2 siRNA significantly lowered the ratio of p-Akt/t-Akt after FGF10 treatment, when compared with both the control group and the negative control siRNA group (Supplementary Figures S2a and d). Besides, FGFR2 knockdown further lowered the ratio of p-Akt/t-Akt without pretreatment of FGF10 (Supplementary Figures S2e and f). These results suggested that FGFR2 mediates FGF10-activation of the PI3K/Akt signaling pathway. Thus, we supposed that the FGFR2/PI3K/Akt signaling pathway is involved in the therapeutic effect of FGF10.

### FGF10 decreases spinal cord tissue damage and motor neuron loss, and promotes locomotor recovery from SCI *in vivo*

**Figure 2:**
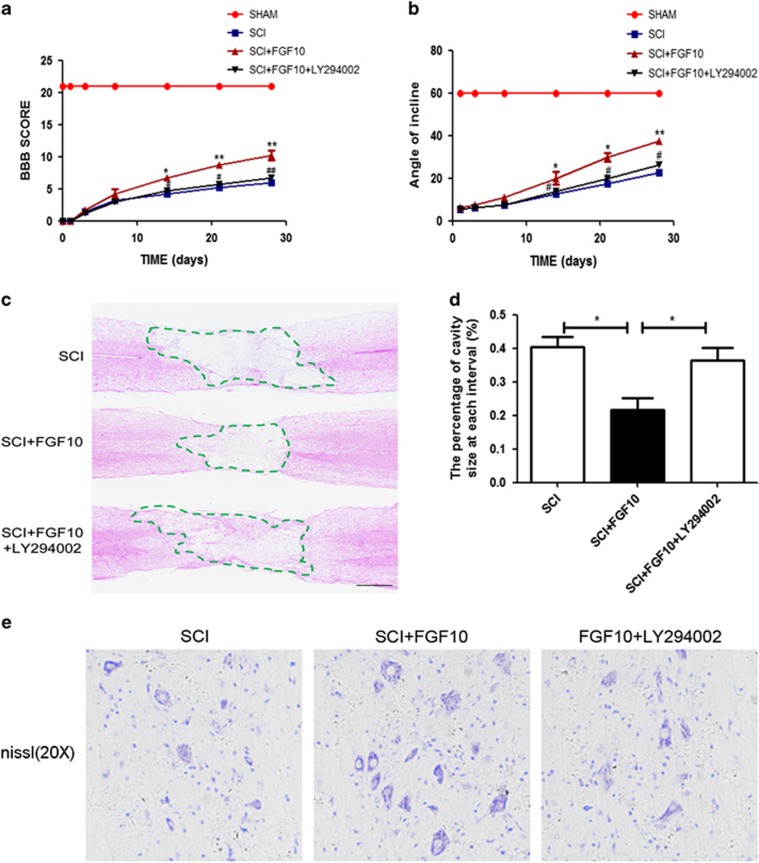
FGF10 decreases spinal cord tissue damage, motor neuron loss and promotes locomotor recovery after acute SCI *in vivo*. (**a** and **b**) The BBB scores and inclined plane test scores of each group. (**c** and **d**) HE staining of each group at 28 days after surgery and quantification data of the percent of cavity necrotic tissue at each interval (scale bar: 200 *μ*m). (**e**) Nissl staining of each group to test the surviving neurons at 28 days after surgery. Data represent the mean±S.D. Significant differences between the treatment and control groups are indicated as *P<0.05, **P<0.01, *n*=5

As spinal FGF10 is increased in neurons after acute SCI, we explored if this could have a therapeutic effect on SCI by administering exogenous FGF10 in SCI model. The therapeutic effect of FGF10 is in part due to activation of the PI3K/Akt pathway in many biological processes, including the early ischemia/reperfusion injury.^23, 25, 26^ BBB scores and inclined plane test scores were using to assess the therapeutic effect of FGF10. BBB scores in the SCI and FGF10 groups were significantly below normal with no significant difference within the first week after surgery. However, BBB scores began to increase at 14 days after surgery in the FGF10 group (Figure 2a). Similarly, we noted higher inclined plane test scores in the FGF10-treated group at 14, 21 and 28 days after SCI (Figure 2b), suggesting that locomotor function was significantly improved compared with the SCI group. To further confirm the neuroprotective role of FGF10, we used a specific PI3K inhibitor, LY294002, in conjunction with FGF10 treatment. LY294002 significantly suppressed beneficial effect of FGF10 on functional recovery (Figures 2a and b). The HE and Nissl staining results revealed that the SCI group displayed greater destruction of central gray matter and peripheral white matter, which followed by remarkable motor neuron loss in the anterior horn. However, the FGF10-treated group had a decreased cavity of necrotic tissue around the injury site and decreased motor neuron loss in the anterior horn, showing that FGF10 protected against severe damage after SCI (Figures 2c and d). Moreover, LY294002 treatment significantly increased the damage caused by SCI, aggravating the cavity of necrotic tissue and decreasing motor neuron survival compared with FGF10 treatment alone (Figures 2c–e). Taken together, FGF10 could exert a neuroprotective effect on SCI *in vivo*.

### FGF10 treatment decreases apoptosis through activation of the PI3K/Akt pathway

**Figure 3:**
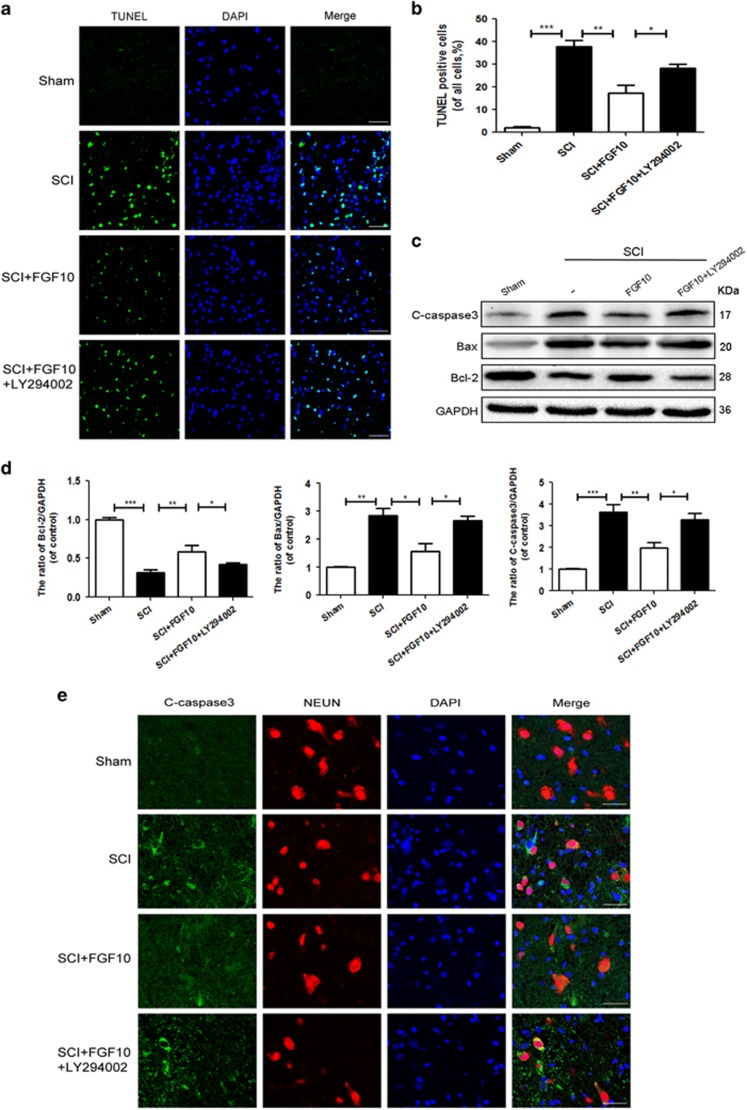
FGF10 reduces apoptosis via activation of the PI3K/Akt pathway. After SCI in rats, (**a** and **b**) TUNEL assay was performed in each group at 7 days after SCI (scale bar: 50 *μ*m). (**c** and **d**) Western blots and quantification data of cleaved-caspase 3, Bax and Bcl-2 of each group at 3 days after surgery. (**e**) Double immunofluorescence of NeuN (red) and cleaved-caspase 3 (green) of each group at 3 days after surgery (scale bar: 50 *μ*m). Data represent the mean±S.D. Significant differences between the treatment and SCI groups are indicated as *P<0.05, **P<0.01, ***P<0.001, *n*=5

To test whether FGF10 treatment decreases apoptosis in SCI, TUNEL staining was performed, SCI significantly increased the number of apoptotic cells compared with the sham group. In comparison, FGF10 treatment greatly reduced apoptotic activity, but this was in part reversed by LY294002 (Figures 3a and b). Moreover, western blot analysis showed increased levels of cleaved-caspase 3 and Bax in the SCI group, which was significantly attenuated by FGF10 treatment. In contrast, FGF10 increased the level of Bcl-2 compare with SCI group. Moreover, LY294002 reversed the anti-apoptotic effect of FGF10 as shown by increased Bax and cleaved-caspase 3 and decreased Bcl-2, which is consistent with our double immunofluorescence staining results (Figures 3c–e). To further investigate the effect of FGF10 on cell viability, we knocked down FGFR2 using siRNA in PC12 cells before FGF10 treatment. For the *in vitro* study, H_2_O_2_ treatment was used to mimic neuronal injury after acute SCI. TUNEL assay results showed that FGFR2 knockdown increased the apoptotic activity compared with the FGF10-treated H_2_O_2_ group (Supplementary Figures S3a and b). On another hand, FGF10 markedly decreased the expression of cleaved-caspase 3 and Bax and increased the expression of Bcl-2 with H_2_O_2_ treatment. However, FGFR2 knockdown reversed the anti-apoptotic effect of FGF10 (Supplementary Figures S3c and d). Similarly, immunofluorescent staining revealed increased cleaved-caspase 3-positive puncta with FGFR2 knockdown compared with the FGF10-treated H_2_O_2_ group (Supplementary Figure S3e). In addition, FGF10 treatment increased the ratio of p-Akt/Akt induced by H_2_O_2_, which was suppressed by FGFR2 knockdown (Supplementary Figures S3f and g,). These results further demonstrated the anti-apoptotic effect of FGF10 after SCI.

### FGF10 improves neurite repair and enhances axonal sprouting in acute SCI

**Figure 4:**
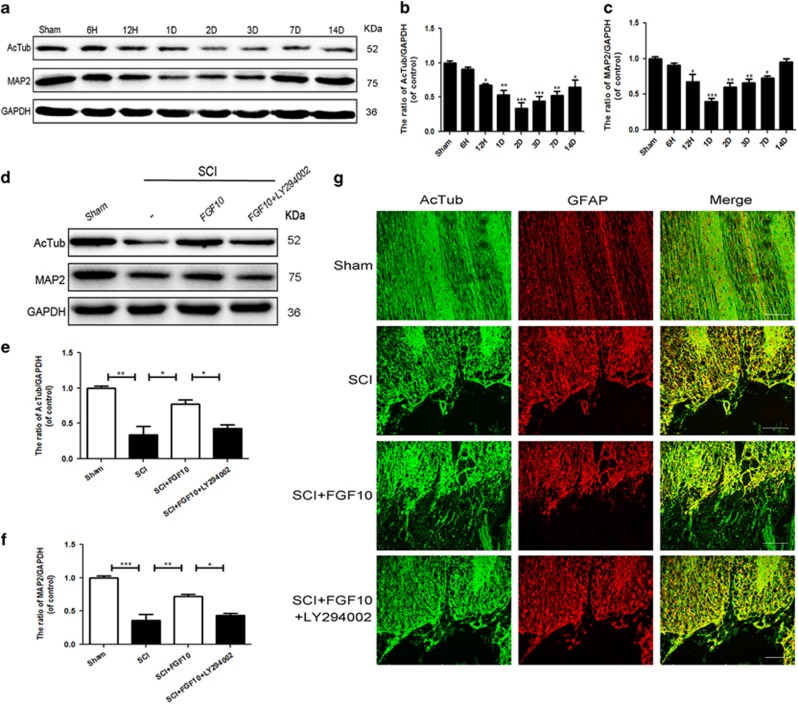
FGF10 promotes neurite repair in acute SCI. (**a**-**c**) Western blots and quantification data of AcTub and MAP2 expression at several time points after acute SCI. (**d**-**f**) Western blots and quantification data of AcTub and MAP2 in each group at 1 day post-surgery. (**g**) Immunofluorescence of AcTub (green) and GFAP (red) of the injured spinal cord sections from tissue in each group at 28 days after surgery (scale bar: 50 *μ*m). Data represent the mean±S.D. Significant differences between the treatment and control groups are indicated *P<0.05, **P<0.01, ***P<0.001, *n*=5

Microtubule-associated protein 2 (MAP2), a specific structural protein in neuron, is mainly expressed in neuronal dendrites and is known to stabilize microtubules and regulate the length of dendrites.^27, 28^ However, the expression of MAP2 and acetylated tubulin (AcTub) after acute SCI is unclear. As shown in Figures 4a and b, the expression of AcTub and MAP2 protein decreased, reaching the lowest point at 1 or 2 days post-SCI (Figures 4a–c), showing that SCI reduced microtubule protein with limited repair capacity. Moreover, AcTub and MAP2 were upregulated in the FGF10 group on the first day after injury compared with the SCI group, but this effect was reversed by LY294002 treatment (Figures 4d–f). Immunofluorescent staining showed that FGF10 treatment promoted the outgrowth of AcTub labeled axons,^29^ which elongate into the distal regions of the SCI area compared with the untreated and LY294002 groups (Figure 4g), suggesting that FGF10 may have a role in stabilizing microtubule structure and repairing neurites after acute SCI. In neuronal cultures, FGFR2 knockdown reversed the increased microtubule stabilization seen with FGF10 treatment by reducing the expression of AcTub and MAP2 (Supplementary Figures S4a–c). Immunofluorescent staining showed that pretreatment with FGFR2 siRNA abolished the beneficial effect of FGF10 on neuronal repair (Supplementary Figures S4d and e). Taken together, FGF10 activated FGFR2/PI3K/Akt signaling contributes to the repair of neurites.

### FGF10 treatment prevents microglia/macrophages activation and reduces pro-inflammatory cytokine release

**Figure 5:**
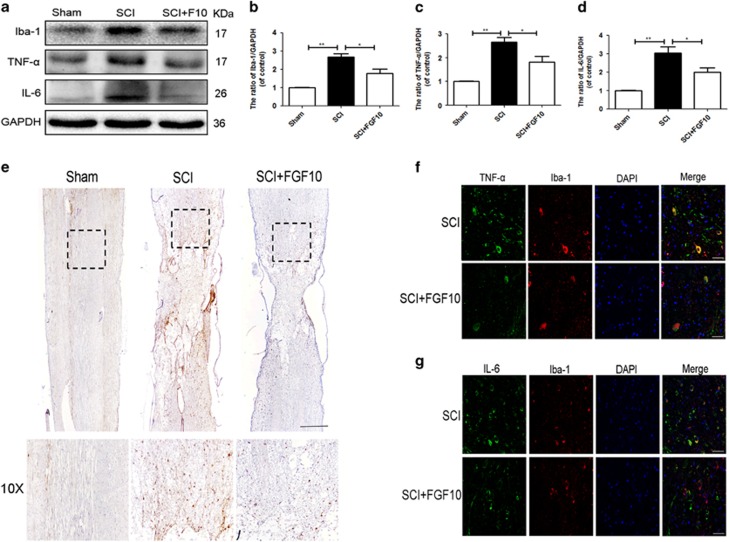
FGF10 prevents microglia/macrophages activation and reduces pro-inflammatory cytokine release in rats after acute SCI. (**a**-**d**) Western blots and quantification data of Iba-1, TNF-*α* and IL-6 in each group at 1 day after SCI. Data represent the mean±S.D. Significant differences between the treatment and SCI groups are indicated as *P<0.05, **P<0.01, *n*=5. (**e**) Immunohistochemical staining of Iba-1 in each group at 1 day after surgery (scale bar: 200 *μ*m). (**f** and **g**) Immunofluorescence of pro-inflammatory cytokines (TNF-*α* and IL-6, green) and Iba-1 (red) in each group at 1 day after surgery

To determine whether FGF10 affected microglia/macrophages activation and pro-inflammatory cytokine release, we examined expression of Iba-1 and pro-inflammatory cytokines IL-6 and TNF-*α*. The SCI group showed increased expression of Iba-1, IL-6 and TNF-*α* compared with sham group, which was significantly reversed by FGF10 treatment (Figures 5a and d). Importantly, the results of immunohistochemical staining of Iba-1 showed FGF10 reduced the Iba-1+ microglia/macrophages population at the injury area (Figure 5e). And double immunofluorescence assay of pro-inflammatory cytokines (TNF-*α*, IL-6) and Iba-1+ cells showed that FGF10 treatment reduced the pro-inflammatory cytokine release in microglia/macrophages after SCI (Figures 5f and g). These results indicated that FGF10 treatment significantly inhibited microglia/macrophages activation and migration, and tightly regulated the production of pro-inflammatory cytokines in microglia/macrophages following SCI.

### FGF10 treatment suppresses the TLR4/NF-*κ*B signaling pathway

**Figure 6:**
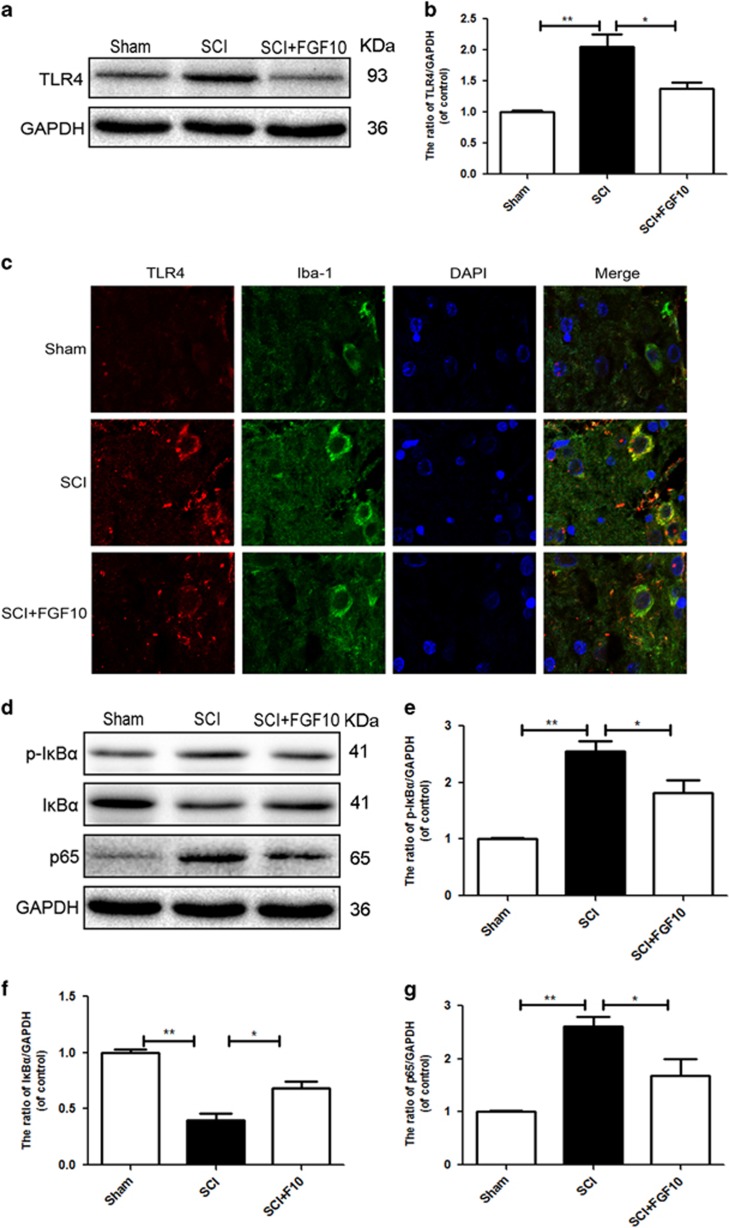
FGF10 suppressed microglia/macrophages TLR4 expression and downstream NF-*κ*B signaling in rats after SCI. (**a** and **b**) Western blots and quantification data of TLR4 in each group at 1 day after SCI. (**c**) Double immunofluorescence staining for Iba-1 positive microglia/macrophages (green) and TLR4 (red) of sections from the tissue at 1 day after SCI. (**d**-**g**) Representative western blots and quantification data of p-I*κ*B*α*, I*κ*B*α* and p65 of each group at 1 day after SCI. Data represent the mean±S.D. Significant differences between the treatment and SCI groups are indicated as *P<0.05, **P<0.01, *n*=5

To further confirm the underlying anti-inflammatory effect of FGF10, we explored whether the TLR4/NF-*κ*B pathway was involved in rats after SCI. We observed increased expression of TLR4 in the SCI group compared with sham group, which was reversed by FGF10 treatment (Figures 6a and b). Immunostaining results showed that FGF10 treatment significantly lowered TLR4-expressing microglia**/**macrophages compared with SCI group (Figure 6c). We also examined the protein levels of p-I*κ*B*α*, I*κ*B*α* and NF-*κ*B (p65). And found higher expression of p65 and p-I*κ*B*α* in the SCI group compared with the sham group, but this was reduced by FGF10 treatment (Figures 6d–g). To further confirm whether the TLR4/NF-*κ*B pathway mediates the anti-inflammatory mechanism of FGF10, we used LPS, a TLR4 ligand, to activate the TLR4/NF-*κ*B pathway in BV-2 cells, which should increase neuroinflammation.^30^ Pretreatment with FGF10 significantly reduced TLR4 expression and attenuated NF-*κ*B activation compared with LPS treatment alone (Supplementary Figures S5a–e). Similarly, immunostaining results showed that pretreatment with FGF10 significantly decreased the amount of TLR4 in LPS-treated BV-2 cells (Supplementary Figure S5f). Nuclear translocation of NF-*κ*B triggers transcription of many inflammatory genes. Immunostaining assays further showed that FGF10 treatment significantly attenuated nuclear translocation of NF-*κ*B induced by LPS (Supplementary Figure S5g).

## Discussion

In recent years, various pharmacological treatments have focused on axonal and dendritic repair to enhance recovery from CNS injury.^31, 32^ Some neurotrophins, including brain-derived neurotrophic factor and nerve growth factor (NGF), have been proven to effectively promote neurite outgrowth.^19, 33^ However, most research neglects the body’s self-repair mechanisms after CNS injury. In this study, we found that endogenous FGF10 is significantly released after SCI from neurons and microglia**/**macrophages, especially in the acute phase. We further characterized the role of endogenous FGF10 after SCI, both in neuron and microglia**/**macrophages.

After crushing SCI, the initial trauma is followed by prolonged secondary injury including many inflammatory, ischemic and neurotoxic events that structurally damage the neuronal integrity around the injury site.^34^ Endogenous ROS activate various intrinsic pathways, including the pro-apoptotic signaling pathways in neurons.^35^ In the 'intrinsic pathway', Bcl-2 family proteins (such as cytochrome c, endonuclease G, caspase and AIF) combine with each other, leading to the release of pro-apoptotic proteins, as well as liberating caspase-activated DNase, triggering activation of the apoptosis.^36, 37^ ROS can activate various upstream signaling mechanisms, including p53 and PI3K/Akt, which both regulate the intrinsic pathway.^38, 39^ The PI3K/Akt pathway is critical for growth and survival in many biological processes, including early ischemia/reperfusion injury as shown in our previous work.^40^ Akt phosphorylates and inactivates Bad, a pro-apoptotic Bcl-2 family protein, reducing apoptosis after cerebral ischemia.^41^ Akt also suppresses the activation of pro-caspase-9, and caspase-9 phosphorylation, preventing apoptotic activation.^42^ The activation of PI3K/Akt signaling pathway by FGF10 could be attributed to FGFR2b, the receptor of FGF10.^43^ Several studies have reported that FGFR2 have a critical role in regulating oxidative stress and cellular apoptosis. In addition, FGFR2 is upregulated in myxoid liposarcoma, and inhibiting the expression of FGFR2 reduced cell proliferation and increased apoptosis.^44^ In this study, we observed higher FGFR2 expression in neurons and significant increase of FGF7 and FGF10 on the first day after acute SCI, which have been reported to activate the FGFR2. So we supposed that FGF10 activated FGFR2/PI3K/Akt signaling may have a role in cellular death. Bcl-2, Bax and cleaved-caspase 3 were used as markers for apoptotic activation or inhibition. Among them, Bcl-2 has an anti-apoptotic effect, whereas the release of Bax and cleaved-caspase 3 are pro-apoptotic.^45^ Our results showed that FGF10 activated the PI3K/Akt pathway and significantly decreased the protein expression of Bax and cleaved-caspase 3, and upregulated the expression of Bcl-2 in rats of SCI. Interestingly, FGFR2 knockdown blocked activation of PI3K/Akt signaling pathway and abolished the anti-apoptotic effect of FGF10. These results showed that FGF10 activated the FGFR2/PI3K/Akt pathway as a neuroprotective mechanism after SCI to reduce neuronal apoptosis caused by oxidative stress.

Moreover, neurons are cells with high energy requirements, and are sensitive to ROS stimulation, especially in their axons and dendrites.^46^ Previous studies including our work noted morphological alterations of neurites, described as bead formation, and reduced number and density of dendrites in ROS-treated granule cells.^47^ One mechanism of ROS-induced neurite degeneration occurs by disruption of cytoskeletal proteins, such as microtubules. Microtubules consist of heterodimers of *α*-tubulin and *β*-tubulin, and are crucial structural components of neurites. Moreover, microtubules have pivotal roles in neuronal function, such anterograde and retrograde transport in the axon.^48^ As acetylated tubulin is abound in stable microtubules, activating histone deacetylase by calcium ions, accelerates microtubule depolymerization through tubulin deacetylation.^49^ Evidence suggests that MAP2 deletion reduces microtubule density and length in dendrites.^50^ In addition, MAP2 mediates a link between cellular signaling and cytoskeletal structure, acting as a molecular scaffold upon which cytoskeleton-modifying proteins dissociate and assemble during neuronal activity.^51^ In this study, we first demonstrated reduced expression of AcTub and MAP2 after acute SCI, directly contributing to neuronal dysfunction and death. Moreover, activating PI3K/Akt signaling has been demonstrated to involve in NGF-induced neurite outgrowth in PC12 cells^52^ and suppressing the MEK/ERK/Akt pathway inhibits neurite outgrowth in N2a cells.^53^ FGF10 has been reported as a morphogen that is a critical for hypothalamic axon growth into the forming median eminence and neurohypophysis.^29^ FGF10 regulates neurogenesis and preserves neurogenic potential through its specific expression pattern in the adult mammalian brain.^54^ In this study, we found that FGF10 activated FGFR2/PI3K/Akt signaling pathway was critical for stabilizing microtubule structure and repairing neurites by regulation the expression of microtubule proteins and outgrowth of AcTub labeled neurites.

We also observed increased expression of FGF10 in microglia/macrophages after SCI, which led us to investigate if FGF10 also is important for microglia/macrophages’ function. After SCI, the prolonged inflammatory response enhances resident microglia/macrophages’ activation and proliferation, which subsequently promotes production of pro-inflammatory factors, such as TNF-*α* and IL-6, creating an inhibitory environment for neurite regeneration.^9^ Activated microglia/macrophages produce a variety of pro-inflammatory mediators, as well as other toxic mediators, which trigger signaling cascades and neurotoxic responses in the secondary phase of SCI. These events significantly contribute to both neuronal death and neurite injury.^55, 56^ Many studies have been reported about the critical role of TLR4 in ischemic CNS. Activation of TLR4 signaling contributes to astrocyte-mediated inflammation, and may control pro-inflammatory astroglial conversion to the neurodegenerative phenotype.^57^ It have been reported to protects blood–brain barrier by inhibiting TLR4-mediated inflammatory pathway in ischemic brain.^58, 59^ Activating microglia/macrophages TLR4 signaling by exogenous or endogenous ligands, such as LPS, heme and fibrinogen, induces nuclear translation of NF-*κ*B, which increases release of pro-inflammatory cytokines and leads to neuronal death.^17, 60^ Strikingly, FGF10 treatment significantly decreased microglia/macrophages activation proliferation, and production of pro-inflammatory cytokines *in vivo*. Using LPS, a TLR4 ligand, to activate downstream signaling, we found that FGF10 treatment was able to decrease TLR4 expression, leading to reduced p-I*κ*B-*α* and I*κ*B-*α* degradation and nuclear translocation of NF-*κ*B transcription factors. These results were consistent with our *in vivo* results, suggesting that the TLR4/NF-*κ*B pathway is involved in the underlying anti-inflammatory mechanism of FGF10.

**Figure 7:**
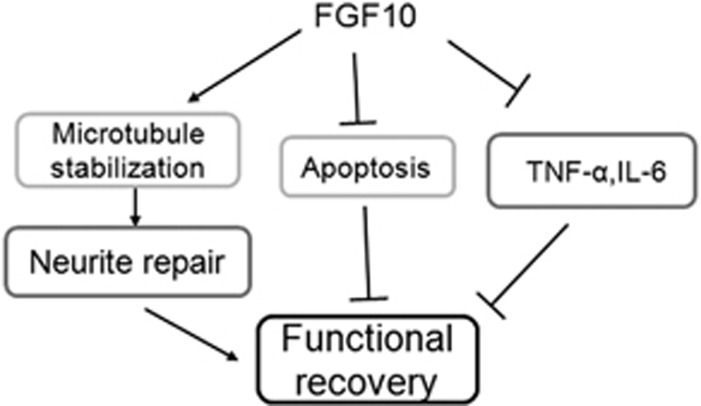
A schematic diagram depicting the potential molecular mechanisms underlying FGF10 protection via neurite repair, reducing apoptosis and decreasing inflammatory cytokines after acute SCI

In conclusion, we first demonstrated that spinal cord-derived FGF10 significantly increased in neurons and microglia/macrophages after acute SCI. Exogenous FGF10 treatment facilitates better functional recovery through the FGFR2/PI3K/Akt signaling pathway, Inhibiting the PI3K/Akt signaling pathway and FGFR2 knockdown abolished these therapeutic effects. Moreover, FGF10 treatment inhibited microglia/macrophages activation and proliferation via regulation of the TLR4/NF-*κ*B pathway, and attenuated the inflammatory response in animals with SCI (Figure 7). As endogenous FGF10 exerts neuroprotective effects following CNS injury, our results suggest that it may in turn be a potentially useful treatment for CNS injury.

## Materials and methods

### Reagents

FGF10 was obtained from Grost (Grost Biotechnology, Zhejiang, China). Antibodies against FGFR2, Bax, Bcl-2, IL-6 and TNF-*α* were purchased from Santa Cruz Biotechnology (Santa Cruz, CA, USA). The MAP2, acetyl-*α*-tubulin (AcTub), TLR4, NF-*κ*B, I*κ*B, p-I*κ*B antibodies and PI3K/Akt inhibitor, LY294002, were obtained from Cell Signaling Technologies (Danvers, MA, USA). The Akt, phosphorylated-Akt (Ser473) and cleaved-caspase 3 antibodies were purchased from Abcam (Cambridge, MA, USA). The reagents of cell culture were obtained from Gibco (Grand Island, NY, USA). All other reagents were purchased from Sigma-Aldrich (St. Louis, MO, USA) unless specified otherwise.

### Surgical procedure

All the surgical interventions and postoperative animal care procedures were in strict accordance with the Animal Care and Use Committee of Wenzhou Medical College. All Sprague–Dawley rats were housed in the SPF Laboratory Animal Room. The rats were injected intraperitoneally with 10% chloral hydrate (3.6 ml/kg), and positioned on a cork platform as discussed previously.^61^ The operator incised the skin to expose the vertebral column in the dorsum, and then performed a laminectomy at the T9 vertebral section. And the spinal cord was clearly exposed and clamped by a vascular clip (30 g force; Oscar, Shanghai, China) for 1 min to simulate a moderate crushing injury model. For the sham group, a T9 laminectomy was performed and the exposed spinal cord for 1 min without compression injury. After surgery, we emptied bladder twice daily until the recovery of bladder function. FGF10 was dissolved in saline and administered intravenously (1mg/kg/day) until the rats were killed.^62^ After surgery, another group of rats was injected with 1mg/kg/day FGF10 and a specific PI3K inhibitor (LY294002, 0.3 mg/kg, i.v.) at the same time. The sham group was injected with saline.

### Cell culture treatment protocols

The PC12 cells and BV-2 cells were obtained from Cell Bank of Type Culture Collection of Chinese Academy of Sciences, Shanghai Institute of Cell Biology, Chinese Academy of Sciences. PC12 cells were cultured in RPMI-1640 medium with 10% (v/v) fetal bovine serum (FBS), 100 U/ml penicillin and 100 U/ml streptomycin. BV-2 cells were cultured in MEM with heat-inactivated 10% (v/v) FBS, 100 U/ml penicillin and 100 U/ml streptomycin. PC12 cells were treated with FGF10 (100 ng/ml) and H_2_O_2_ (100 *μ*M) for 8 h. BV-2 cells were treated with FGF10 (100 ng/ml) and LPS (0.5 *μ*g/ml) for 24 h. All experiments were performed at least three times.

### Locomotion recovery assessment

To assess the locomotion recovery in rats after SCI, the Basso, Beattie and Bresnahan (BBB) scores and the inclined plane test were used as mentioned previously.^61^ In short, the BBB scores range from 0 point (complete paralysis) to 21 points (normal locomotion) according to the muscle strength and joint movement of rats. Concurrently, rats were evaluated in two positions (right side or left side up) on a testing device. For each position, a rat could keep its position for 5 s without falling was recorded. In this study, BBB scores and the inclined plane test were performed by two blinded independent researchers at several time points after surgery.

### Hematoxylin–eosin (HE) and nissl staining

To measure the cavity area of spinal cord tissue in each group after surgery, rats in all groups were killed at 28 days and the spinal cord tissues were embedded in paraffin. Longitudinal sections (5 mm thick) were cut into 5-*μ*m thickness for HE staining. Transverse sections were incubated in 1% cresyl violet acetate for Nissl staining to measure the surviving neurons.

### Western blot assay

Spinal cord tissue from T7 to T10 was collected at 1 day and 3 days after surgery. Briefly, spinal cord tissue and cells were lysed using RIPA with phosphatase inhibitors and protease inhibitors cocktail and then protein concentration was measured by bicinchoninic acid reagents (Thermo, Rockford, IL, USA), equivalent amounts of protein was separated with 8–12% SDS–PAGE gels, and transferred to polyvinylidene fluoride membranes (Bio-Rad, Hercules, CA, USA). Following blocking with 5% nonfat milk, the primary antibodies were incubated: anti-MAP2 (1:500), anti-acetyl-*α*-tubulin (1:1000), anti-FGFR2 (1:200), anti-cleaved-caspase 3 (1:500), anti-NeuN (1:1000), anti-GFAP (1:1000), anti-Iba-1 (1:500), anti-TLR4 (1:500), anti-GAPDH (1:1000), anti-NF-*κ*B (1:400), anti-I*κ*B (1:400) and anti-p-I*κ*B (1:400), followed by their respective secondary antibodies. The bands were detected by the ChemiDicTM XRS + Imaging System (Bio-Rad), and the intensity of these bands were analyzed using Image Lab 3.0 software (Bio-Rad). Experiments were performed at least three times.

### Immunofluorescence staining

Transverse and longitudinal sections (5-*μ*m thick) were deparaffinized and rehydrated. PC12 cells were fixed with 4% PFA for 1 h. And tissue and cells slices were blocked by 5% bovine serum albumin (BSA) for 30 min. Then, they were incubated with the following primary antibodies overnight: anti-acetyl-*α*-tubulin (1:1000), anti-FGFR2 (1:200), anti-cleaved-caspase 3 (1:500), anti-NeuN (1:1000), anti-GFAP (1:1000), anti-Iba-1 (1:500), anti-TLR4 (1:400) and anti-NF-*κ*B (1:400). The next day, the following secondary antibodies were incubated for 1 h: Alexa-Fluor 488 donkey anti-mouse/rabbit, Alexa-Fluor 647 donkey anti-mouse/rabbit. Then, the slices labeled with DAPI for 7 min. All images were observed using a Nikon ECLIPSE Ti microscope (Nikon, Tokyo, Japan).

### Immunohistochemical staining

Transverse and longitudinal sections (5-*μ*m thick) were deparaffinized, rehydrated and then blocked by addition of 3% (v/v) H_2_O_2_ for 10 min followed by incubation in 5% BSA for 30 min. After incubation with primary antibodies (anti-FGF1, 2, 7, anti-Iba-1), the samples were incubated with the respective second antibodies and counterstained with hematoxylin. Images were obtained using a light microscope.

### The TUNEL method

To test apoptotic DNA fragmentation, transverse sections were removed at 4–5 mm rostral and caudal of the lesion, and TUNEL staining was performed 7 days after SCI. The tissues (5 *μ*m thick) were deparaffinized, and rehydrated. Cells were incubated with 4% PFA for 1 h. Then, tissues and cells were incubated with 0.1 % Triton X-100 for 30 min. Apoptotic cells of spinal cord tissue were stained with *In Situ* Cell Death Detection Kit (Roche Molecular Biochemicals, Basel, Switzerland) according to the manufacturer’s instructions, and DAPI. All apoptotic changes were tested under a Nikon ECLIPSE Ti microscope (Nikon).

### Small interfering RNA transfection

PC12 cells were treated with FGFR2 siRNA (100 pmol, GeneChem, Shanghai, China) in serum-free medium containing Lipofectamine 2000 (Life Technologies, Carlsbad, CA, USA). After transfection for 6 h, medium was switched to medium containing 5% FBS for 24 h. After treatments, cells were harvested for further experiments.

### Statistical analysis

The results are expressed as the mean±S.D. from at least three independent experiments. And statistical significance was analyzed using Graphpad Prism (La Jolla, CA, USA) (one-way analysis of variance (ANOVA) and Tukey’s test). *P*<0.05 was considered statistically significant.
