# Supplementary figures and images for: Neuron and microglia/macrophage-derived FGF10 activate neuronal FGFR2/PI3K/Akt signaling and inhibit microglia/macrophages TLR4/NF-κB-dependent neuroinflammation to improve functional recovery after spinal cord injury
Source: Cell Death Dis. 2017 Oct 5;8(10):e3090–. doi: 10.1038/cddis.2017.490 (PMC5682656; doi:10.1038/cddis.2017.490)

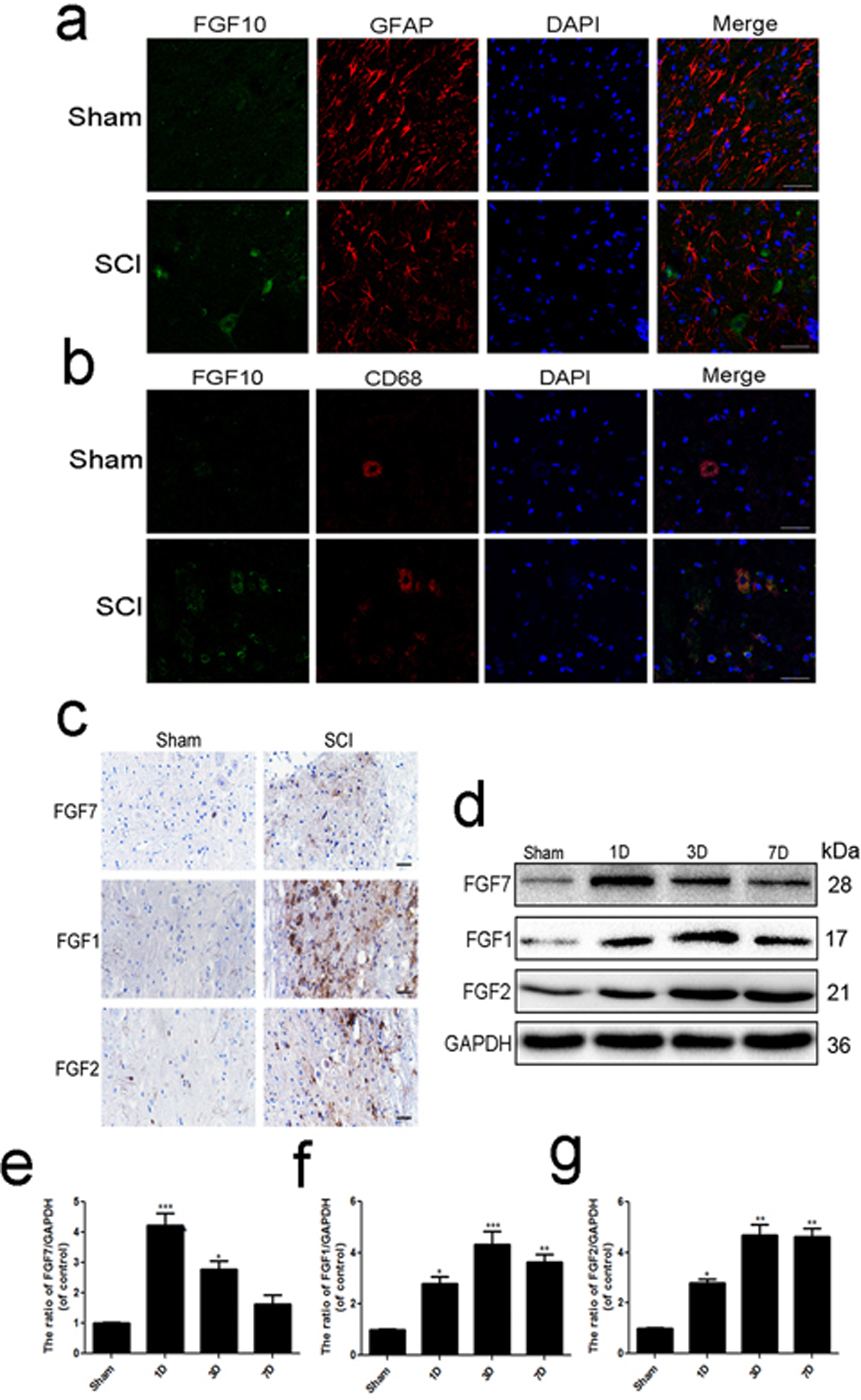

Supplement: Supplementary Figure S1 [file cddis2017490x1.tif]

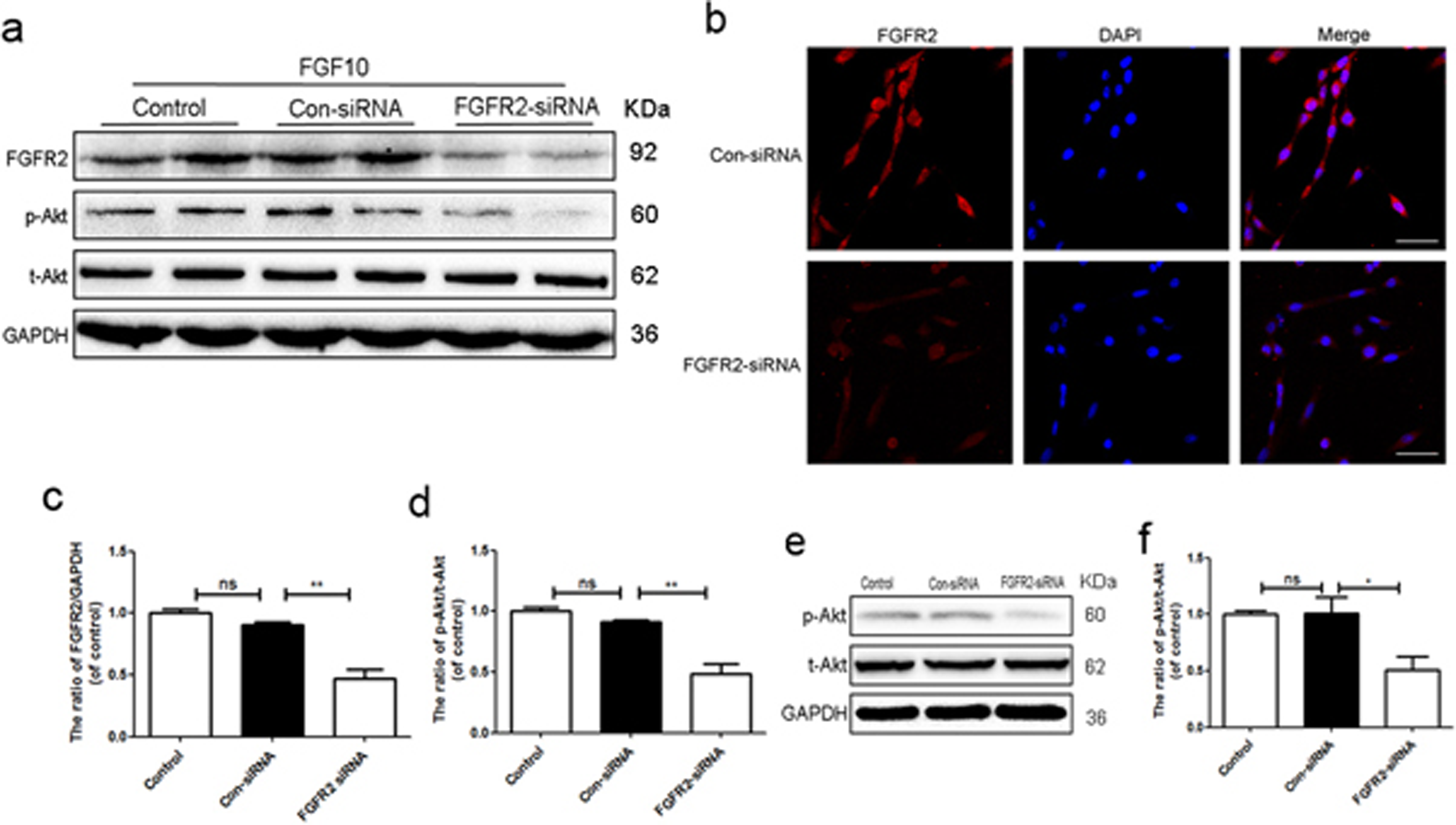

Supplement: Supplementary Figure S2 [file cddis2017490x2.tif]
