# Supplementary material for: Neuron and microglia/macrophage-derived FGF10 activate neuronal FGFR2/PI3K/Akt signaling and inhibit microglia/macrophages TLR4/NF-κB-dependent neuroinflammation to improve functional recovery after spinal cord injury
Source: Cell Death Dis. 2017 Oct 5;8(10):e3090–. doi: 10.1038/cddis.2017.490 (PMC5682656; doi:10.1038/cddis.2017.490)
Supplement: Supplementary Figure Legends [file cddis2017490x6.doc]

**Supplementary Figure Legends**

**Supplementary Figures S1 The expression of FGF10 in astrocyte and CD68+ microglia**/macrophages, **and the expression of other FGF (1, 2, 7) after SCI**. (a and b) Double immunofluorescence of FGF10 and cellular markers for (a) astrocyte (GFAP) or (b) microglia/macrophages (CD68), in spinal cord tissue adjacent to lesion (scale bar: 50μm). (c) Immunohistochemical staining of FGF7, FGF1 and FGF2 in each group at 1 day after surgery (scale bar: 50μm). (d-g) Western blots and quantification data of FGF7, FGF1 and FGF2 of each group. Data represents the mean ± S.D. Significant differences between the treatment and SCI groups are indicated as *P<0.05, **P<0.01, ***P<0.001, n=5.

**Supplementary Figures S2** **FGFR2 knockdown markedly attenuated FGF10-activated PI3K/Akt signaling in PC12 cells**. PC12 Cells were treated with negative control siRNA (con-siRNA) or FGFR2 siRNA before FGF10 treatment (1 100 nmol). (a, c and d) Representative Western blots and quantification data of FGFR2, p-Akt and Akt in the PC12 cells as treated above, and each group is shown with two bands. (b) Immunofluorescence staining of FGFR2 (red) of each group. (e and f) Representative Western blots and quantification data of p-Akt and Akt in the PC12 cells as treated above. Data represents the mean ± S.D. Significant differences between the treatment and SCI groups are indicated as *P<0.05, **P<0.01, n=5.

**Supplementary Figures S3** **FGFR2 knockdown attenuated the antiapoptotic effect of FGF10 induced by H_2_O_2_ *in vitro***. (a and b) TUNEL assay was used to assess the apoptosis of each group in neuronal cultures. (c-d) Western blots and quantification data of cleaved caspase 3, Bax and Bcl-2 in each group. (e) Caspase 3 (red) was detected by immunofluorescence staining combined with DAPI staining for nuclei in PC12 cells. (f-g) Representative Western blots and quantification data of p-Akt and Akt in each group. Data represents the mean ± S.D. Significant differences between the treatment and SCI groups are indicated as *P<0.05, **P<0.01, ***P<0.001, n=5.

**Supplementary Figures S4 FGFR2 knockdown reverses the ability of FGF10 to preserve microtubule protein and repair neurite**. (a-c) Western blots and quantification data of AcTub and MAP2 of each group. (d-e) Immunofluorescent staining of AcTub (green) was performed to assess the neurite length in each group. Data represents the mean ± S.D. Significant differences between the treatment and SCI groups are indicated as *P<0.05, **P<0.01, ***P<0.001, n=5.

**Supplementary Figures S5 FGF10 inhibits LPS-induced TLR4/NF-κB signaling pathway activation *in vitro***. (a-e) Western blots and quantification data of TLR4, p-IκBα, IκBα and p65 in each group of BV2 cells. (f-g) Immunofluorescence staining for TLR4 (red) and p65 (green) in each group of BV2 cells. Data represents the mean ± S.D. Significant differences between the treatment and SCI groups are indicated as *P<0.05, **P<0.01, ***P<0.001, n=5.
